# Stomach blowout post binge-eating episode: a case report

**DOI:** 10.1093/bjrcr/uaaf052

**Published:** 2025-11-26

**Authors:** Rutger J Lensing, Peter-Paul Teunisse, Annemarie C M Bellegem, Karin Horsthuis

**Affiliations:** Department of Radiology, Amsterdam UMC, University Hospital, Amsterdam, The Netherlands; Department of Psychiatry, Amsterdam UMC, University Hospital, Amsterdam, The Netherlands; Department of Radiology, Amsterdam UMC, University Hospital, Amsterdam, The Netherlands; Department of Psychiatry, Amsterdam UMC, University Hospital, Amsterdam, The Netherlands; Department of Radiology, Amsterdam UMC, University Hospital, Amsterdam, The Netherlands; Department of Psychiatry, Amsterdam UMC, University Hospital, Amsterdam, The Netherlands; Department of Radiology, Amsterdam UMC, University Hospital, Amsterdam, The Netherlands; Department of Psychiatry, Amsterdam UMC, University Hospital, Amsterdam, The Netherlands

**Keywords:** gastric perforation, gastric dilatation, binge-eating, blowout

## Abstract

Acute gastric dilatation is a rare but serious condition that can lead to ischemia, necrosis, and perforation of the stomach. This case report describes a 21-year-old female patient with an eating disorder who developed acute gastric necrosis and perforation following a binge-eating episode. A 21-year-old female with a history of eating disorder not otherwise specified presented to the referring hospital with severe abdominal pain, and on physical examination, the suspicion of subcutaneous emphysema. Chest radiography showed the subcutaneous emphysema and also revealed a pneumothorax and a possible pneumomediastinum. Her condition deteriorated, prompting a CT scan that showed extensive pneumomediastinum, subcutaneous emphysema, a massively distended stomach, and portal venous air. The patient was transferred to our hospital, where further imaging confirmed these findings. After further deterioration and a suspected perforation, a second CT scan was performed, confirming a gastric perforation with extensive free fluid in the abdomen. An exploratory laparotomy revealed gastric perforation with necrosis and peritonitis, necessitating a sleeve gastrectomy. The patient underwent successful surgery with resection of necrotic gastric tissue and sleeve gastrectomy. Postoperative recovery was uncomplicated, and follow-up showed no further complications. Early surgical intervention was crucial in managing this life-threatening condition. Acute gastric dilatation and subsequent necrosis are rare but potentially fatal complications in patients with eating disorders. Prompt recognition and surgical intervention are essential to reduce morbidity and mortality.

## Clinical presentation

A 21-year-old female presented to the referring hospital with severe abdominal discomfort following a binge-eating episode 3 days prior. The patient, likely suffering from an eating disorder not otherwise specified (EDNOS), was found to have subcutaneous emphysema upon physical examination, with stable vital parameters.

The initial chest X-ray confirmed subcutaneous emphysema and revealed mediastinal free air and no free air subdiaphragmatically, leading to a diagnosis of pneumothorax. Her condition worsened in the evening, with increased abdominal pain and the onset of tachycardia. A CT scan of the chest and abdomen showed extensive free air in the mediastinum, subcutaneous emphysema, a massively distended stomach, air in the portal vein branches, and traces of free fluid in the abdomen, but no free air intra-abdominally. A nasogastric tube was inserted, and the patient was subsequently transferred to our hospital for further treatment.

**Figure 1.**
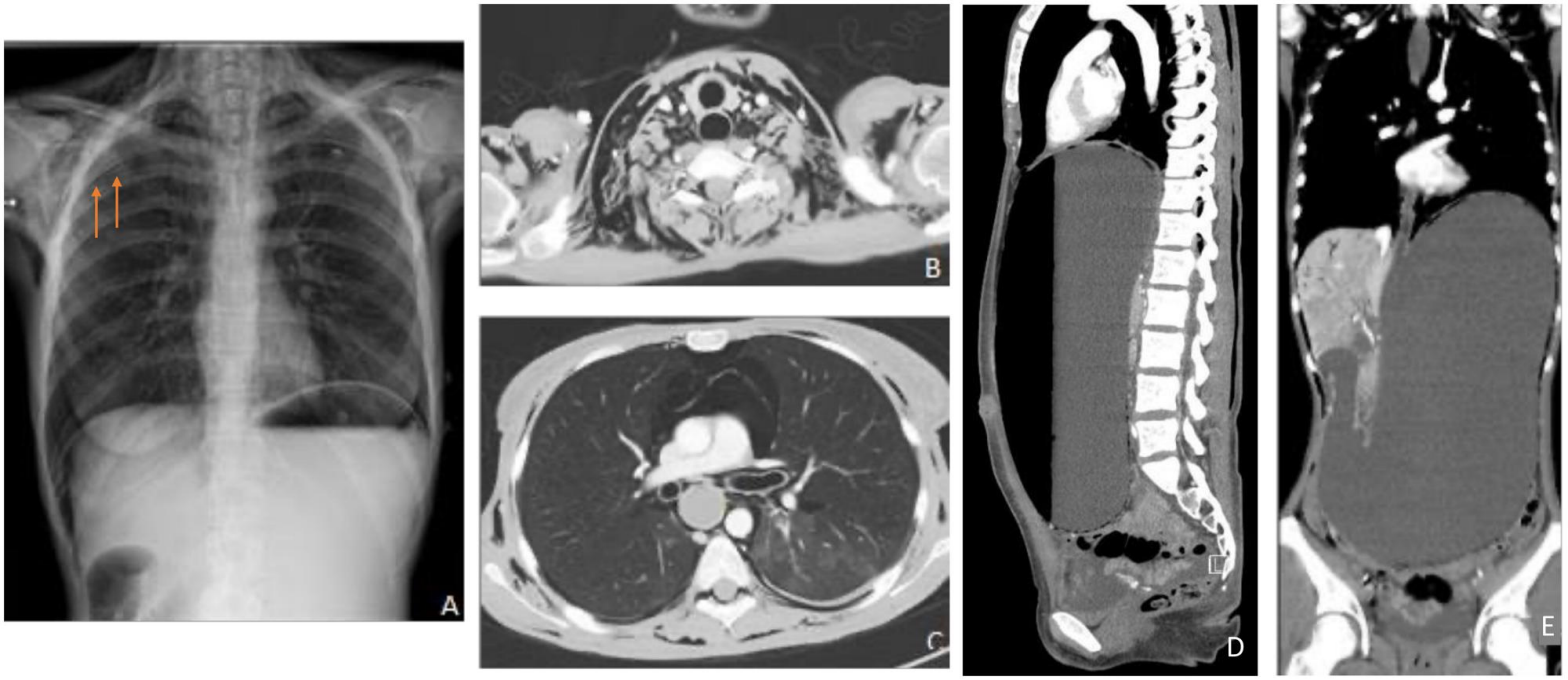
(A) Chest X-ray showing subcutaneous emphysema and a pneumothorax (indicated by orange arrows). (B) Extensive subcutaneous emphysema in the neck and mediastinum. (C) showing the pneumomediastinum. (D) and (E) sagittal and coronal reconstruction of the extensively filled stomach, with a maximum diameter of 34 cm, as well as air in the portal veins.

Retrospective review of the initial CT revealed subtle pneumatosis along the distal oesophageal wall (Figure 4), as well as within the gastric fornix and body (Figure 1), together with small foci of gas in the portal veins. Findings consistent with early gastric ischemia.

Upon arrival at our emergency room, the patient had extensive crepitus, tachypnoea (40 breaths per minute), and tachycardia (150 bpm). Her abdomen was tense and painful, though no additional abnormalities were initially diagnosed.

## Image findings

### Chest X-ray

**Figure 2.**
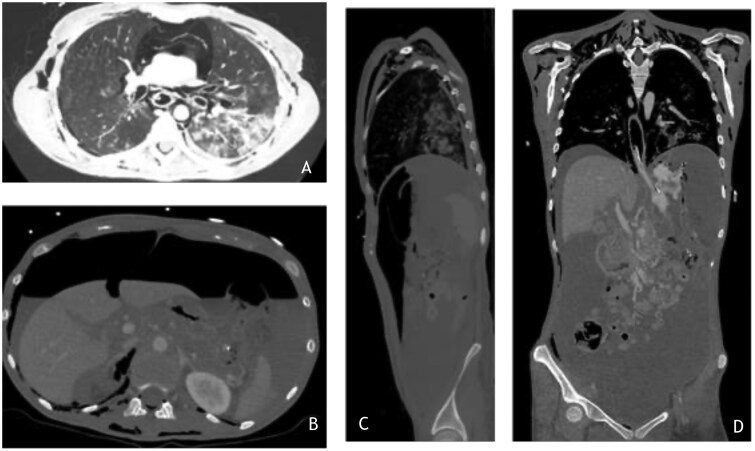
(A) Chest CT in a lung widows demonstrating subcutaneous emphysema, a tiny right pneumothorax and pneumomediastinum. In the left lung base consolidation due to aspiration. (B) Abdominal image showing extensive free air and peritoneal fluid, due to the ischaemic stomach blowout. (C) Sagittal reconstruction of the CT-abdomen again showing the free air, subcutaneous emphysema and the aspiration pneumonia. (D) coronal reconstruction partially depicting the nasogastric tube and the free peritoneal fluid, as well as pneumatosis in the fornix of the stomach.

The chest X-ray showed subcutaneous emphysema and highlighted free air below the diaphragm. A wider bore nasogastric tube was inserted, draining 2 L of content. Due to clinical deterioration, another CT scan with intravenous and oral contrast was performed (**Figure 2**).

### CT Chest and Abdomen

- Contrast visible in the distal oesophagus and stomach antrum.
- In contrast with the previous CT, a non-dilated stomach.
- Extensive pneumatosis located outside the stomach, free air intra-abdominally extending into the pelvis.
- Significant free fluid in all abdominal quadrants, likely originating from the gastric contents. No evident hyperdense extraluminal contrast visualized. But it was thought to be highly diluted with the free fluid.
- Normal calibre of the small intestine and colon.
- Pneumomediastinum and bilateral pneumothorax.
- Subcutaneous emphysema extending from the skull base to the perineum.
- Peribronchovascular consolidations in the lower left lobe and, to a lesser extent, in the lower right and upper left lobes.
- Traces of gas in the portal branches.

## Treatment

**Figure 3.**
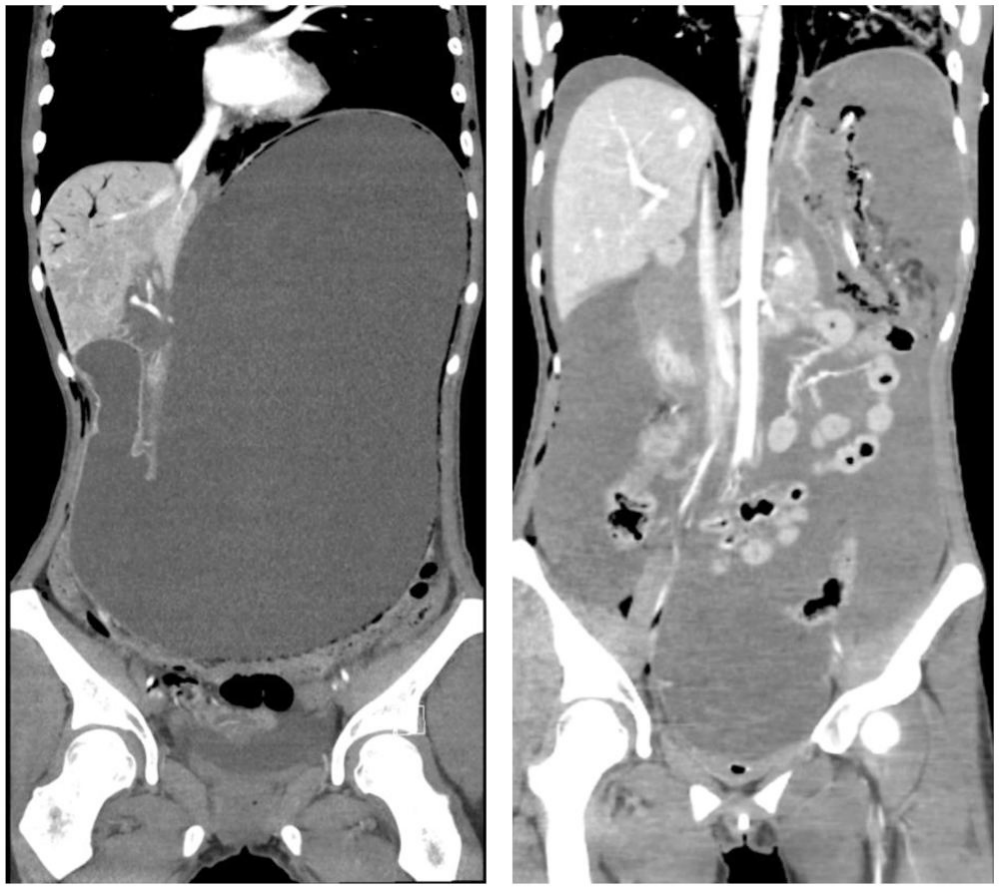
Coronal reconstruction. Left shows the initial CT, pre-perforation, showing pneumatosis the wall of the gastric fornix, adjacent to the lung, and in the gastric body, projecting within in the pelvis. On the right, the second CT is shown, acquired after deterioration with a collapsed stomach after perforation and pneumatosis in the gastric fornix.

Given the deterioration and the CT findings, an exploratory laparotomy was performed (Figure 3). Upon incision, fluid and air were released under pressure, with 3 L of stomach contents being aspirated from the peritoneal cavity, followed by extensive peritoneal lavage.

**Figure 4.**
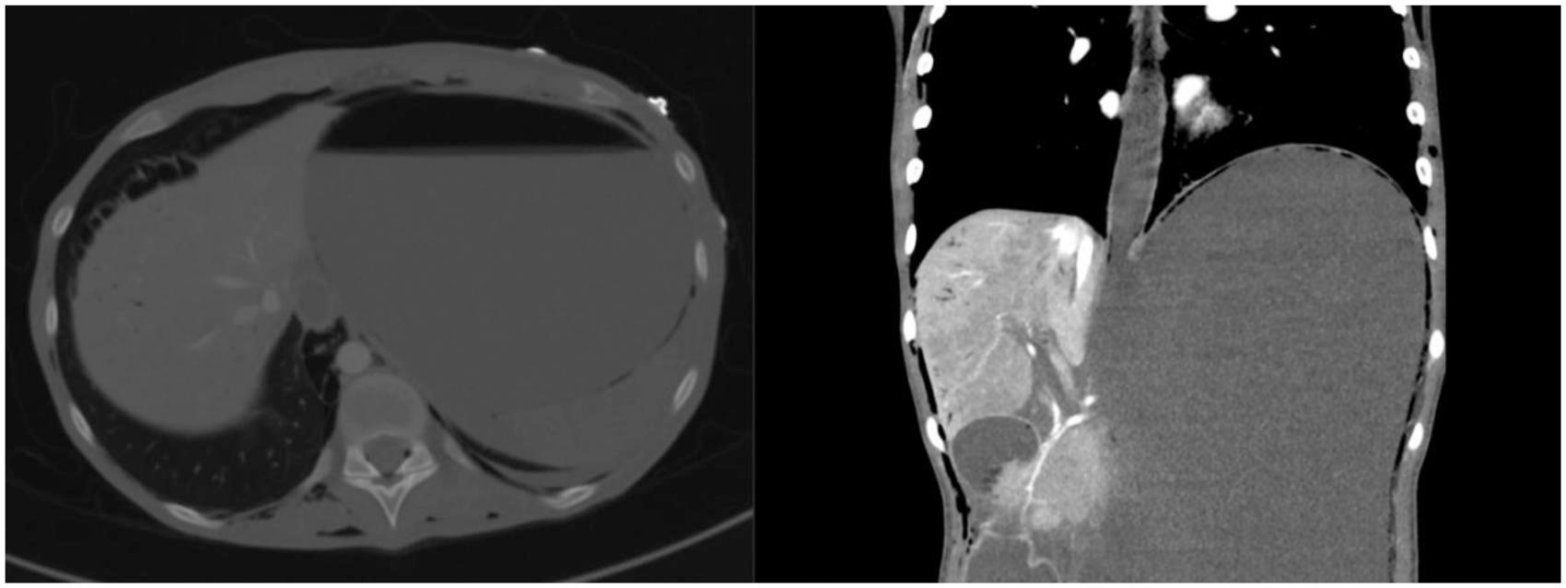
Axial and coronal reconstruction of the gastro-oesophageal junction shows subtle pneumatosis within the distal oesophageal wall, gastric fundus, in the portal veins, extrapleural and subcutaneously. These findings likely represent early ischemic involvement, preceding gastric perforation.

Inspection revealed a rupture of the stomach, secondary to ischemia of the fundus and corpus. The lesser curvature of the stomach retained sufficient blood supply, allowing for a sleeve resection. A gastroenterologist performed an intraoperative gastroscopy to rule out ischemia or perforation of the oesophagus, with neither found.

## Outcome

The patient underwent a sleeve resection following the gastric rupture. Postoperatively, she was initially supported with enteral feeding through a feeding tube and received psychiatric care. Her recovery was uncomplicated, and she was discharged home after 20 days in good condition.

## Discussion

Gastric dilatation was first described in 1833 by S.E. Duplay. Various causes of gastric dilatation range from mechanical (eg, pyloric stenosis, volvulus) to non-mechanical (eg, gastroparesis, eating disorders). Acute gastric dilatation (AGD) presents with abdominal pain, distension, and vomiting. Acute gastric necrosis is rare due to the stomach’s rich blood supply.^1^^,^^2^ Several reports document gastric dilatation in anorexic or bulimic patients, but fewer describe acute perforation due to ischemia or necrosis. Immediate surgical intervention is critical due to the high mortality and morbidity associated with delayed treatment.^3^

The pathophysiology of gastric necrosis is debated. One theory suggests that atrophy of gastric musculature in bulimic patients, due to periods of prolonged starvation, leads to an inability to empty contents during binge-eating episodes. Prolonged dilatation and increased intraluminal pressure result in insufficient oxygenation and subsequent necrosis and perforation, causing peritoneal contamination, sepsis, and abscess formation.^2^^,^^4^^,^^5^

Ultrasound is widely used in emergency medicine for a rapid assessment of various intra-abdominal pathology. Conventional ultrasound can detect free intraperitoneal fluid, pneumoperitoneum and even signs of bowel perforation. More advanced ultrasound techniques, such as microvascular flow imaging (MVFI), detecting ischemia and intra-abdominal free air, can also be used.^6^ Microvascular flow imaging is a high-resolution Doppler technique that enhances the visualization of low-velocity blood flow in small vessels. In gastric ischemia, MVFI may demonstrate diminished vascularity or even absent flow in the affected areas. This could provide early indications of necrosis. However, if the perforation has already occurred, MVFI has limited value. CT remains superior for assessing the air distribution and extraluminal contents.^7^

In the Netherlands, ultrasound is primarily utilized for Focused Assessment with Sonography for Trauma (FAST) in emergency settings, where its primary objective is to detect free intraperitoneal fluid. Given the sudden deterioration of the patient, the suspected perforation and the complexity of this case, we deemed a CT scan—as the gold standard—as the most appropriate imaging modality, as it provides superior visualization of both ischemic changes and associated complications such as pneumothorax, pneumomediastinum, and portal venous air. This approach ensured a timely and accurate diagnosis.

Conservative management with a nasogastric tube can initially reduce intraluminal pressure. If the patient’s condition deteriorates, CT is the fastest diagnostic tool. In cases of ischemic injury, prompt laparotomy is necessary.

Our patient experienced a rupture of the greater curvature without necrosis of the lesser curvature, which is less common than ruptures of the lesser curvature (8.3% vs 36.1%).^8^ Resection of necrotic tissue and sleeve gastrectomy were successful.

The literature is heterogeneous regarding the anatomical site most predisposed to ischemia or perforation in AGD. Several case reports describe preferential involvement of the greater curvature and fundus, theorized to be due to increasing wall tension with increasing expansion (also known as Laplace’s law) and relative venous congestion.^1^^,^^5^

Conversely, other studies—particularly those focusing on peptic ulcer-related perforation—report the lesser curvature as a common site, potentially due to differences in elasticity and vascular anatomy.^9^^,^^10^ In our case, the involvement of the greater curvature is in keeping with the mechanical and vascular mechanisms proposed for distention-related ischemia.

The precise timing of the gastric perforation in our patient cannot be determined with certainty. However, based on the clinical course and available imaging, it is likely that the perforation occurred prior to the patient’s arrival in our emergency department. It is plausible that the gastric wall was already ischemic due to prolonged distension and increasing intraluminal pressure. This ischemia could have weakened the integrity of the gastric wall, predisposing it to rupture under even minimal additional stress, such as the transfer process or increased abdominal tension upon presentation to our emergency room. Although the nasogastric tube inserted at the initial hospital may have provided temporary decompression, it did not halt the underlying pathophysiological progression toward perforation.

Retrospectively, the initial CT scan likely already demonstrated early gastric pneumatosis, because of the pneumatosis throughout the wall of the entire stomach. The most probable explanation for the development of a pneumothorax, pneumomediastinum, and air in the portal venous branches is that these findings preceded the gastric perforation and resulted from forceful vomiting and increased intragastric pressure. This mechanism could also explain the pulmonary consolidations, which were most likely secondary to aspiration.

Similarly, the presence of portal venous gas can be attributed to elevated intragastric pressure due to gastric dilatation and excessive vomiting. Under these conditions, air may have translocated through the compromised mucosa into the submucosa and mesenteric venous circulation, subsequently reaching the portal venous system. The process may have been facilitated by underlying gastric call alterations, such as ischemia and early necrosis, which could have increased mucosal permeability and predisposed the stomach for further complications.^11^^,^^12^
